# Abundance, origin, and phylogeny of plants do not predict community‐level patterns of pathogen diversity and infection 

**DOI:** 10.1002/ece3.6292

**Published:** 2020-05-18

**Authors:** Robin Schmidt, Harald Auge, Holger B. Deising, Isabell Hensen, Scott A. Mangan, Martin Schädler, Claudia Stein, Tiffany M. Knight

**Affiliations:** ^1^ Department of Community Ecology Helmholtz‐Centre for Environmental Research–UFZ Halle (Saale) Germany; ^2^ German Centre for Integrative Biodiversity Research (iDiv) Halle‐Jena‐Leipzig Leipzig Germany; ^3^ Institute of Biology/Geobotany and Botanical Garden Martin Luther University Halle‐Wittenberg Halle (Saale) Germany; ^4^ Institute of Agricultural and Nutritional Sciences, Phytopathology and Plant Protection Martin Luther University Halle‐Wittenberg Halle (Saale) Germany; ^5^ Tyson Research Center & Department of Biology Washington University in St. Louis St. Louis Missouri USA; ^6^ Department of Biology and Environmental Sciences Auburn University at Montgomery Montgomery AL USA

**Keywords:** Enemy release hypothesis, exotic species, host abundance, phylogenetic community context, plant–fungal interactions, temperate grasslands

## Abstract

Pathogens have the potential to shape plant community structure, and thus, it is important to understand the factors that determine pathogen diversity and infection in communities. The abundance, origin, and evolutionary relationships of plant hosts are all known to influence pathogen patterns and are typically studied separately. We present an observational study that examined the influence of all three factors and their interactions on the diversity of and infection of several broad taxonomic groups of foliar, floral, and stem pathogens across three sites in a temperate grassland in the central United States. Despite that pathogens are known to respond positively to increases in their host abundances in other systems, we found no relationship between host abundance and either pathogen diversity or infection. Native and exotic plants did not differ in their infection levels, but exotic plants hosted a more generalist pathogen community compared to native plants. There was no phylogenetic signal across plants in pathogen diversity or infection. The lack of evidence for a role of abundance, origin, and evolutionary relationships in shaping patterns of pathogens in our study might be explained by the high generalization and global distributions of our focal pathogen community, as well as the high diversity of our plant host community. In general, the community‐level patterns of aboveground pathogen infections have received less attention than belowground pathogens, and our results suggest that their patterns might not be explained by the same drivers.

## Graphical abstract

We investigated the effects of plant abundance, origin, and phylogenetic community context on the diversity and infection levels of aboveground pathogens in a temperate grassland in the central United States. We found no relationship between host abundance, plant origin or phylogeny, and our observed infection levels. However, exotic host species harbored a more generalist pathogen community.

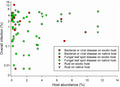

## 1 INTRODUCTION

Pathogens can dramatically influence plant fitness and are thereby assumed to mediate patterns of community assembly, such as plant species coexistence, plant invasions, and dominance (Alexander, 2010; Bever, Mangan, & Alexander, 2015). To better understand these processes, we need to identify the factors that determine pathogen diversity and infection in plant communities. While most of the literature on host–pathogen patterns has focused on one or a few host plants and their interactions (e.g., Krupinsky, Bailey, McMullen, Gossen, & Kelly Turkington, 2002), there is a growing literature examining community‐level patterns of pathogen diversity and infection (Massoni et al., 2019; Mommer et al., 2018; Vaz et al., 2018; see also review by Mishra, Hättenschwiler, & Yang, 2020). Three main factors are commonly invoked to explain patterns of pathogen diversity and infection across plant species in a community (Mordecai, 2011): (a) host plant abundance (Connell, 1971; Janzen, 1970; Parker et al., 2015), (b) host plant origin (Klironomos, 2002; Mitchell & Power, 2003), and (c) plant defense traits (Holt & Dobson, 2006; Viola et al., 2010).

Pathogens can respond positively to increase in abundance of their host and subsequently decrease host population growth rates, thereby providing an advantage to rare host species yet to be heavily infected (Gilbert, 2005 ;Jarosz & Davelos, 1995). This negative frequency dependence caused by pathogens has been hypothesized to be one of the dominant stabilizing mechanisms that maintain plant species coexistence (Bever, 2003; Chesson, 2000; Connell, 1971; Janzen, 1970; Mangan et al., 2010). Indeed, both the incidence and severity of pathogen infection have been shown to be positively related to host abundance in natural communities (Burdon & Chilvers, 1982; Parker et al., 2015), but see Spear and Mordecai (2018). If multiple potential pathogens for a given host species exist, the probability of co‐infection by more than one species of pathogen could increase with host abundance (Burdon & Chilvers, 1982; Seabloom et al., 2015). However, empirical studies testing for a relationship between plant abundance and pathogen diversity are scarce (Moore & Borer, 2012;Mundt & Browning, 1985) or test the relationship indirectly by modifying diversity and relative abundances of plant species (Mommer et al., 2018). Furthermore, interactions between co‐infecting pathogens and differences across studies in the degree of specialization of pathogens make it difficult to synthesize results across studies (Ostfeld, Glass, & Keesing, 2005; Seabloom et al., 2015; see review by Benítez, Hersh, Vilgalys, & Clark, 2013).

Exotic plant species are expected to exhibit lower pathogen species diversity and disease severity than native species because they left behind many of their native pathogens when introduced into a new area (Elton, 1958; Keane & Crawley, 2002; Williamson, 1996). The enemy release hypothesis further posits that low pathogen infection allows exotic plants to have increased fitness and a competitive advantage against enemy‐regulated native species, thereby contributing to invader establishment and spread (Hierro, Maron, & Callaway, 2005). Results from broad‐scale studies support the enemy release hypothesis by showing that plants are attacked by more pathogen species in their native compared to their naturalized ranges (Mitchell & Power, 2003). However, pathogen species are known to accumulate on exotic plants over time (Flory, Alba, Clay, Holt, & Goss, 2018; Stricker, Harmon, Goss, Clay, & Luke Flory, 2016), and some studies have even shown more pronounced pathogen attacks on exotic plants than on their native relatives (Colautti, Ricciardi, Grigorovich, & MacIsaac, 2004).

According to the enemy release hypothesis, reduced pathogen attack on naturalized plants should result from the absence of specialists pathogens (Keane & Crawley, 2002). The source of infection for naturalized plants should be pathogens that are generalized in their host range and/or those that have cosmopolitan geographic distributions and are thus present in both the native and naturalized range of plants (Parker & Gilbert, 2004; Prospero & Cleary, 2017). Indeed, exotic plant species have been shown to be released from pathogens with a restricted geographical distribution (van Kleunen & Fischer, 2009). A so far untested prediction is whether pathogens with a restricted geographical distribution, regardless of their host specificity, infect exotic hosts to a lower extent than native hosts.

Plant morphological and chemical traits, including those determining resistance against natural enemies, are often phylogenetically conserved (Farrell, 2001), and thus, poorly defended plants that have high diversity of pathogens should cluster together on a phylogeny creating phylogenetic signal in the diversity of pathogens found on plants (Clark & Clegg, 2017). Closely related plants are likely to be attacked by the same pathogen species (Gilbert & Webb, 2007), and thus, evolutionarily distinct plant species in a community might be attacked by fewer pathogen species. Indeed, it has long been hypothesized that evolutionary distinct species might have an advantage when colonizing new habitats because they will not share enemies with the resident community (Darwin's Naturalization Hypothesis, Cadotte, Campbell, Li, Sodhi, & Mandrak, 2018; Darwin, 1872). Therefore, the relationship between pathogen infection and host abundance or origin may be masked by the phylogenetic affiliations of species within a community (Parker et al., 2015).

Interactions between phylogeny, abundance, and origin should determine the diversity and infection of pathogens observed on plant species in a community. For example, it is possible that native but not exotic plants suffer from high pathogen diversity and infection levels when they occur at high densities, if the enemy release hypothesis results in low pathogen diversity and infection for exotic plants regardless of their abundance (Keane & Crawley, 2002). We are not aware of any studies that have simultaneously considered how all three factors influence patterns of pathogen diversity, incidence, and severity on plant species in a community. Additionally, aboveground pathogens have rarely been considered as drivers of grassland plant community structure and composition in grasslands (Allan, Ruijven, & Crawley, 2010; Borer, Hosseini, Seabloom, & Dobson, 2007; Parker & Gilbert, 2018; Spear & Mordecai, 2018). Here, we report the results of an observational study conducted on 51 host species on three sites in a temperate grassland in the central United States to test the following predictions for patterns of foliar, stem, and floral pathogen infection:

1. Effects of host abundance: If pathogens can respond positively to increases in the abundance of their host, pathogen infection should increase with the abundance of host species.
2. Effects of native versus exotic host origin: If plant species leave specialized enemies behind when introduced to a new range, exotic plant species should display less pathogen diversity and infection compared to native species. Furthermore, pathogens restricted to North America, being either specialists or generalists, might infect exotic invaders to a lower extent than native hosts.
3. Effects of phylogeny: If defense traits are phylogenetically conserved, we expect a phylogenetic signal in the diversity of pathogen groups and levels of infection on plant species.

Our focal pathogens include a wide range of taxa, including downy mildews, rusts, fungal leaf spot diseases, bacteria, and viruses. These taxa differ broadly in their traits and thus might also differ in their community‐level patterns. Thus, we considered how “pathogen group” interacted with “host abundance” and “host origin,” to determine patterns of pathogen infection.

## 2 METHODS

### 2.1 Study area

The field sites were located at Washington University's Tyson Research Center (38°31’N, 90°33’W), located near St. Louis, Missouri, USA. The climate is temperate with 914 mm annual precipitation. The cherty, stony, and well‐drained soils of the Gasconade‐Clarksville‐Menfro Association dominating this area were formed in chert‐free limestone residuum, cherty limestone residuum, and deep loess (Benham, 1982). These soils rest on bedrock primarily composed of Mississippian limestone (Burlington‐Keokuk formation) and Devonian sandstones (Bushberg sandstone). The vegetation in this region mainly consists of Oak‐Hickory forest, interspersed with patches of old fields, which are marked by the dominance of several species of perennial grasses and tall‐growing Asteraceae (https://tyson.wustl.edu/).

### 2.2 Study design

For our study, we selected three sites separated from each other by at least 1.2 km. At each site, we documented plant community composition by collecting information on the identity and percent cover of all plant species in 20 1m^2^ plots placed in a grid design that spanned the site (plots separated by 3–10 m, depending on site size). From this species pool, we sampled pathogens on all vascular plant species that contained at least 10 individuals at a site and at our time of sampling (July 2015, *N* = 51 total plant species of which 40 are native and 11 exotic). A threshold of 10 individuals allowed adequate sample size for statistical power while also allowing relatively rare species to be included in our observations. Plant species abundance ranged from 0.02 to 11.4 percent cover on a particular site (see Appendix A1‐3). Exotic species showed the same typical lognormal distribution in abundance as native species did, with a few very abundant species and many species with less than one percent cover.

Plant individuals were randomly chosen by blindly throwing a pencil in the surrounding vegetation and subsequently picking the individual of the respective species closest to the fallen pencil. We sampled 10–15 individuals per plant species at each site. For each individual, pathogen infection apparent on the surface of stems, leaves, and flowers was assessed separately using a percentage‐based nine‐level rating scheme, according to Oberforster (2001), ranging from 1 = 0% infection to 9 ≥ 70% infection. Additionally, we estimated pathogen infection for all aboveground parts for each individual, using the same rating scheme. As floral infections were rare (*N* = 2 infected individuals of *Festuca subverticillata*) and the results for foliar and stem surface infection rates were similar to those based on all aboveground parts, we only present results for all aboveground parts.

We assigned all pathogens observed on individual plants to broad pathogen groups following Rottstock, Joshi, Kummer, and Fischer (2014): fungal leaf spot diseases, powdery mildews, rusts, and downy mildews. While the “fungal leaf spot disease” group is not a taxonomically defined group, species in this group produce morphologically similar disease symptoms, which allows for accurate attribution of infection signs. We recorded rust and downy mildew infection as presence of sporulation structures, powdery mildews as presence of mycelium, and fungal leaf spot diseases as presence of necrotic leaf lesions (Rottstock et al., 2014). We chose this as our measure of pathogen infection, because loss of photosynthetic tissue is a direct indicator of pathogen impact on host productivity and directly comparable across host species (Parker & Gilbert, 2007;Parker et al., 2015). Although being a major pathogen group, we did not find any smut fungi on our plants. However, we also found a considerable amount of infection signs not relatable to the mentioned fungal, or fungal‐like, pathogen groups, but which we could attribute to be caused by pathogenic bacteria or plant viruses (henceforth called “bacterial and viral diseases”). While infection patterns may differ broadly between bacteria and viruses and among groups of fungal pathogens (García‐Guzmán & Heil, 2014;Rodriguez‐Moreno, Ebert, Bolton, & Thomma, 2018), our goal was to cover infection patterns over a broad range of pathogen groups, and disease of bacterial and viral origin constituted a considerable proportion of the visible infection signs in our community. Distinct morphological and life‐history characteristics across these pathogen groups allow pathogen groups for each host plant to be documented in the field.

Most pathogens can not be directly identified to species in the field. However, to attain an entire pathogen species list for each host plant species across all sites, we took one sample of each occurring sign of pathogen infection for each plant species and site, which we dried and pressed to preserve them for further determination via digital microscopy (VHX‐2000, Keyence Corp.). Digital microscopic imaging extends conventional microscopy by combining the power of optical imaging, electronic detection, and computerized analysis (Chen, Zheng, & Liu, 2011), thereby providing the opportunity to efficiently conduct pathogen identification on a single device. Taxonomy and determination of pathogens to species followed Farr, Bills, Chamuris, and Rossman (1989), Brandenburger (1985), Klenke and Scholler (2015), and MycoBank, the online database of the International Mycological Association (Crous, Gams, Stalpers, Robert, & Stegehuis, 2004).

### 2.3 Pathogen diversity and infection

At each site, we quantified pathogen diversity with two metrics: the total number of pathogen groups for each host species (“pathogen groups per species”) and the mean absolute number of pathogen groups per plant individual (“number of pathogen groups per plant individual”). For each site and pathogen group, we quantified pathogen infection with three metrics: the percentage of infected individuals per plant species (“incidence”), the mean percentage of infected plant tissue per plant species (“severity”), and the product of both (“overall infection”). Moreover, we calculated the incidence, severity, and overall infection across all pathogen groups to estimate total pathogen load for each host species (“total incidence,” “total severity” and “total overall infection,” see Table 1, also see Rottstock et al. (2014)).

**Table 1:** Definition of response variables used in text

Response variables	Description
Pathogen groups per plant species	Total number of pathogen groups per plant species and site
Number of pathogen groups per individual	Mean absolute number of pathogen groups per plant individual
Pathogen incidence	Percentage of infected individuals per plant species, site, and pathogen group
Pathogen severity	Mean percentage of infected plant tissue per plant species, site, and pathogen group
Overall infection	Product of pathogen incidence and pathogen severity per plant species, site, and pathogen group
Total incidence	Incidence across all pathogen groups per plant species and site
Total severity	Sum of severity across all pathogen groups per plant species and site
Total overall infection	Sum of overall infection across pathogen groups per plant species and site

### 2.4 Plant phylogeny

We used a dated molecular phylogeny of vascular plants (Zanne et al., 2014) to create a phylogeny for our 51 focal plant species (Appendix C). Our focal species that were missing from this tree were bound into the phylogeny at the genus level using the function congeneric.merge in the pez package of R (Pearse et al., 2015).

### 2.5 Pathogen specialization and distribution

For each pathogen identified to species level, we calculated the mean number of host genera and families, as a measure of host range, by extracting the number of known host genera and families from the USDA Fungus‐Host distribution database (https://nt.ars‐grin.gov/fungaldatabases/fungushost/fungushost.cfm). We restricted our metric to these higher taxonomic levels, since the number of known host species is unavailable for some of our pathogen species. We used the same database to extract the geographic distribution for each pathogen.

### 2.6 Statistical analysis

We used Blombergs K (Blomberg, Garland, & Ives, 2003) to test for phylogenetic signal of pathogen diversity, incidence, severity, and overall infection across plant species. For plant species occurring on multiple sites, values of pathogen diversity and infection were averaged across sites. Blombergs K was computed along with an associated p‐value by comparing the real association between each pathogen response variable and phylogeny to a null distribution obtained by random permutations of the data. *P*‐values less than .05 indicate nonrandom patterns (i.e., phylogenetic signal). We found no evidence of a phylogenetic signal in any pathogen response variable (see Results) and thus did not consider phylogeny in any other statistical analyses.

Statistical analysis on the effects of host abundance and origin on pathogen diversity and infection was performed using generalized linear mixed models in SAS**®** Release 9.4 (procedure GLIMMIX). We analyzed the total number of pathogen groups using a model with Poisson error distribution and a logarithmic link‐function. The dataset for the number of pathogen groups per individual was square root‐transformed (Ahrens, Cox, & Budhwar, 1990) and analyzed using models with Gaussian error distribution and identity link‐function. For the response variables incidence and total incidence, we used models with binomial error distribution and a logit link‐function. The percentage data for severity, total severity, overall infection, and total overall infection were logit‐transformed (Warton & Hui, 2011) and analyzed using models with Gaussian error distribution and identity link‐function. Plant species occurring on more than one site were treated separately for each site. In each analysis, “site” (*N* = 3 sites) was considered as a random effect and “host abundance” (*N* = 18 species for the Perilla site, *N* = 23 species for the Carduus site, and *N* = 20 species for each site) and “host origin” (*N* = 2 categories), as well as their interaction, were treated as fixed effects. We considered “pathogen group,” as well as its interactions with “host abundance” and “host origin,” as a fixed factor in the analyses examining incidence, severity, and overall infection.

To test for differences in pathogen specialization between exotic and native hosts, we used a Mann–Whitney U test with host origin (“exotic” and “native”) as predictor variable and the mean number of host genera (and families, respectively) for each pathogen species as response variable (procedure NPAR1WAY). To compare the geographic distribution of pathogens (“restricted to North America” vs. “distributed on more than one continent”) between exotic and native host plants, we performed a chi‐square test for independence (procedure FREQ).

## 3 RESULTS

### 3.1 Pathogen community

Of the 51 sampled plant species across three sites, 46 showed visible signs of pathogen infection (Appendix A1‐3). In total, we found one downy mildew, three powdery mildew, seven rust, and 27 fungal leaf spot diseases; among them, three species not yet reported on the respective host in the United States. From these 38 pathogen infections, we could identify 24 to species, which included 15 fungal leaf spot diseases (mainly from the genera *Cercospora* and *Septoria*), seven rusts, and two powdery mildews (Appendix B). Most (23) pathogen species visibly infected only one out of 51 host species, potentially reflecting high local host specificity (Appendix B). An additional 36 cases of pathogen infection remained unidentified and were attributed to bacterial and viral diseases (Appendix A1‐3).

### 3.2 Effects of host phylogeny on pathogen diversity and infection

Our 51 host species represent a broad range of angiosperms, including both monocots and dicots (Appendix C). There was high variation across these plants in their pathogen incidence (ranging from 0% to 100%), severity (ranging from 0.1% to 14.3%), and overall infection (ranging from 0% to 12.6%, data not shown). Despite this, we found no evidence of a phylogenetic signal in any pathogen response variable (Table 2).

**Table 2:** Results of the phylogenetic analyses measuring the strength of phylogenetic signal of pathogen diversity, incidence, severity, and overall infection among the plant species found on our three study sites. For incidence, severity, and overall infection, pathogen groups were analyzed separately. Powdery mildews (3 plant species) and downy mildews (1 plant species) were not considered because of low sample size

Pathogen group	Response variable	*N*	Blomberg's K	*p*‐value
All	Pathogen groups per species	51	0.075	.321
Bacterial and viral diseases	Incidence	51	0.114	.124
	Severity	30	0.142	.121
	Overall infection	51	0.092	.374
Fungal leaf spot diseases	Incidence	51	0.025	.824
	Severity	27	0.247	.422
	Overall infection	51	0.035	.774
Rusts	Incidence	51	0.188	.165
	Severity	8	0.688	.441
	Overall infection	51	0.136	.348

### 3.3 Effects of host abundance on pathogen diversity and infection

**Figure 1:**
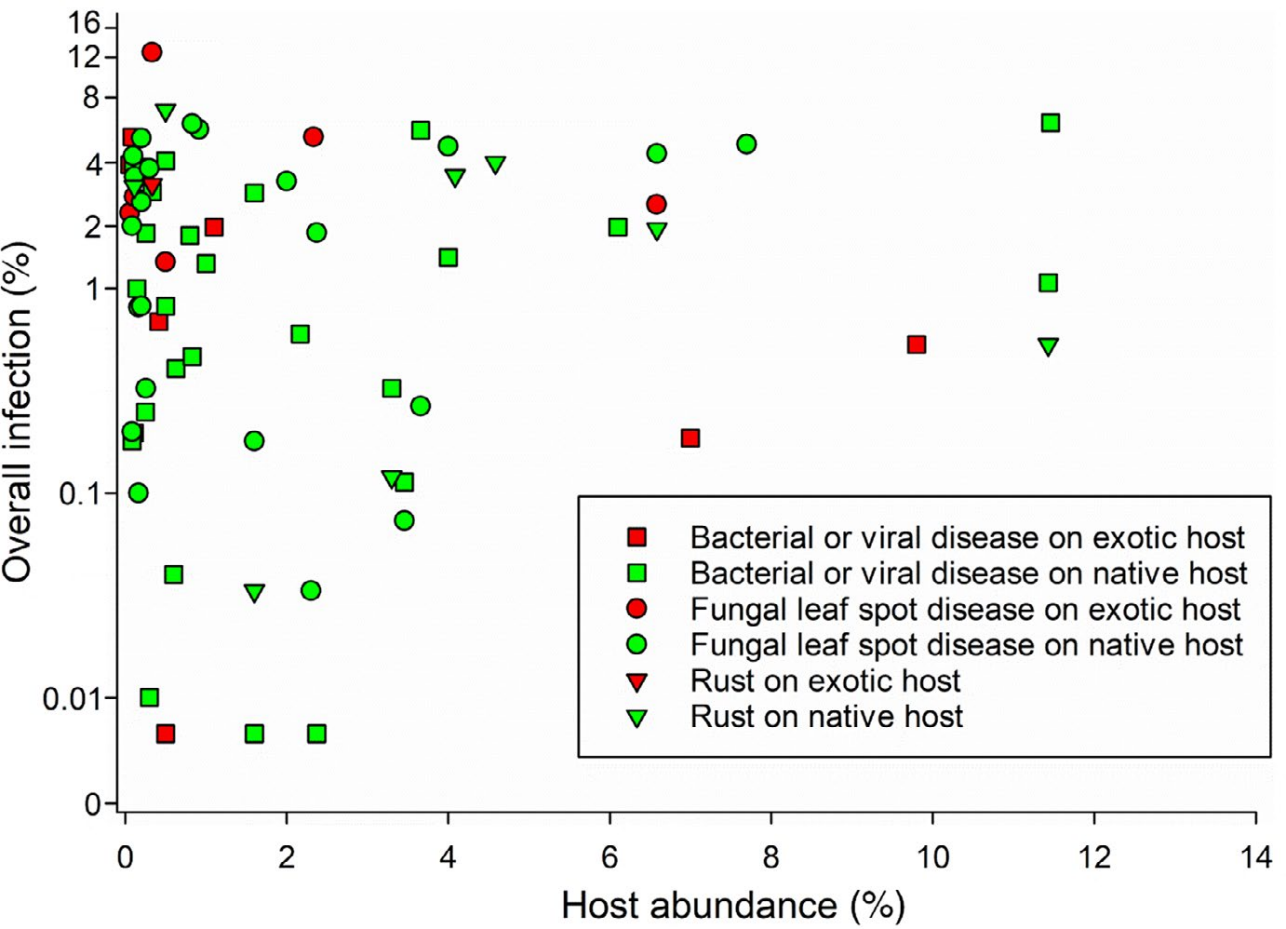
Effect of host abundance (percent cover) on overall infection by different pathogen groups on native and exotic hosts. Each symbol represents a host species with its corresponding abundance and mean overall infection levels across all infected individuals and sites. Please note that downy and powdery mildews were excluded due to low sample size

Host abundance did not influence pathogen diversity nor any of the three metrics of pathogen infection (Table 3 and Figure 1). The incidence, severity, and overall infection differed across pathogen groups, but this effect was only significant for overall infection (Figure 1 and Table 3). There was also no effect of host abundance on total incidence, total severity, or total overall infection (Table 3).

**Table 3:** Summary of results of generalized linear mixed models for the effects of host abundance, origin, and pathogen group, as well as their interactions, on pathogen diversity, pathogen infection, and overall infection levels. Study site was included as a random effect and did not have statistically significant effects on any response variable. Please note that downy and powdery mildews were excluded from all analyses considering “pathogen group,” due to low sample size. Hyphens (“‐”) indicate analyzes that were not performed, and see methods section for further details. For description of terms, see Table 1

	*Host abundance [A]*	*Host origin [O]*	*Pathogen group [PG]*	*A x O*	*A x PG*	*O x PG*	*A x O x PG*
Pathogen diversity	*F*‐values						
*D.f.*	1, 55	1, 55	‐	1, 55	‐	‐	‐
Pathogen groups per species	0.12	1.95	‐	2.19	‐	‐	‐
Number of pathogen groups per individual	0.22	0.80	‐	0.22	‐	‐	‐
Pathogen infection	*F*‐values						
*D.f.*	1, 169	1, 169	2, 169	1, 169	2, 169	2, 169	2, 169
Incidence	0.11	0.09	1.95	0.15	1.08	0.12	0.10
*D.f.*	1, 58	1, 58	2, 58	1, 58	2, 58	1, 58	1, 58
Severity	0.21	0.87	2.10	1.19	0.24	0.18	0.18
*D.f.*	1, 169	1, 169	2, 169	1, 169	2, 169	2, 169	2, 169
Overall infection	0.40	1.51	5.56**	2.15	0.49	0.22	0.04
Total infection	*F*‐values						
*D.f.*	1, 55	1, 55	‐	1, 55	‐	‐	‐
Total incidence	0.28	0.81	‐	0.05	‐	‐	‐
Total severity	0.15	0.82	‐	2.86	‐	‐	‐
Total overall infection	0.11	2.00	‐	2.37	‐	‐	‐

* *p* < .05.

** *p* < .01.

*** *p* < .001.

### 3.4 Effects of host origin on pathogen diversity and infection

The origin of plant species did not significantly influence any metric of pathogen diversity or infection (Table 3). Exotic host species showed similar levels of diversity (1.31 vs. 1.19 pathogen groups per species in exotic vs. native hosts), number of pathogen groups per individual (0.87 vs. 0.78 pathogen groups per plant individual), incidence (18% vs. 16%), severity (3.9% vs. 2.7%), and, consequently, overall infection (0.7% vs. 0.5%) compared to native host species.

### 3.5 Effects of host origin on pathogen specialization and distribution

**Figure 2:**
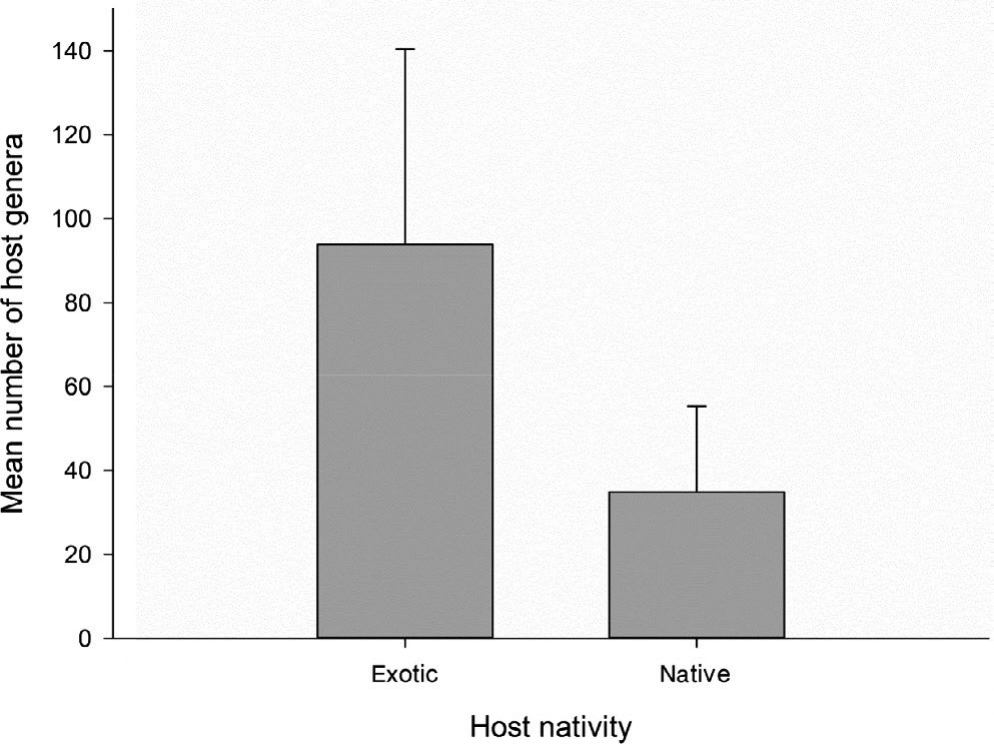
Mean number of host genera (and standard error) for pathogen species infecting exotic (*n* = 7) and native (*n* = 17) host plants on our three study sites. A Mann–Whitney U test showed that pathogen species found on exotic hosts have a greater reported host range than pathogen species on native hosts: Z = 2.194 with *p* = .028 (two‐tailed)

The pathogen species found on exotic plant species had a greater reported host range (mean number of host genera) compared to pathogens infecting native hosts (Figure 2). This relationship was marginally significant when the mean number of reported host families was considered (24 vs. 11.4 reported host families, *p* = .0561). The geographic distribution of pathogens (pathogens restricted to North America vs. pathogens distributed on more than one continent) did not significantly differ between exotic and native hosts (Table 4).

**Table 4:** Total number of pathogen species of large geographic distribution (more than one continent, according to the USDA Fungus‐Host distribution database) and species whose distribution is restricted to North America (NA), on native and exotic hosts. A chi‐square test showed that pathogen distribution and host origin are distributed independently: χ^2^ = 0.33 with *p* = .57

	Pathogen species with a large geographic distribution	Pathogen species with a distribution restricted to NA	
Pathogen species on native hosts	13	4	17
Pathogen species on exotic hosts	6	1	7
	19	5	

## 4 DISCUSSION

At our study sites in grasslands in the central United States, we observed frequent infection of almost all sampled plant species by pathogens (>90%). Yet, these observed infection levels were not related to either host abundance, origin, or phylogeny. Pathogens are expected and often observed to have higher infection on more abundant hosts, creating a stabilizing mechanism that allows rare plants to persist in diverse plant communities (Alexander, 2010; Bever et al., 2015; Mangan et al., 2010; Mordecai, 2011). However, it has also been shown that diverse natural communities favor multihost pathogens, thereby decoupling the frequency of pathogen infection from the abundance of individual host species (May, 1991; Spear, Coley, & Kursar, 2015). It is not yet fully understood how the degree of pathogen specialization and its role in promoting species coexistence varies among different pathogen groups (Mordecai, 2011). While most of our pathogen species were only observed on a single plant species, these pathogen species are known to attack multiple genera of plants. Our results suggest these multihost pathogens are able to infect both rare and common plants, and observations might depend on the abundance of the pathogen species in the community (many of which are likely to be rare) and on pathogen transmission rates.

Overall infection levels differed across pathogen groups, which are unsurprising, given differences in pathogen life cycles and transmission mechanisms (Deacon, 2006). Rusts, which showed the lowest overall infection levels of all pathogen groups, are often specialists and dependent on an alternate host for sexual development (Leung & Kot, 2015). Fungal leaf spot diseases, which showed the highest infection levels of all pathogen groups, usually have no host shifts during their life cycle and are mostly generalists with broad host ranges (Deacon, 2006). Among bacteria and viruses, there are species with host shifts and species without host shifts. Similarly, the degree of specialization also varies widely across these broad groups of plant pathogens, which demonstrated intermediate levels of overall infection.

We find that many of our pathogen species that are known to attack many genera of hosts are only observed to infect a small proportion of their potential hosts in our plant community. In particular, fungal leaf spot diseases infected only few of their possible hosts in our focal communities (see Appendix B). Several mechanisms might explain this. First, many of the pathogen species may have low transmission rates (Gilbert & Levine, 2017). Second, the dilution effect, a reduction in disease levels observed when host species diversity is high and when hosts vary in their susceptibility to a pathogen (Civitello et al., 2015; Ostfeld & Keesing, 2000), can cause patterns of high local host specificity of pathogens (see also Rottstock et al., 2014). Temperate grasslands are among the most species‐rich plant communities (Wilson, Peet, Dengler, & Pärtel, 2012), and for soil‐borne (Maron, Marler, Klironomos, & Cleveland, 2011; Schnitzer et al., 2011) and foliar (Mitchell, Tilman, & Groth, 2002) pathogens, it has been shown that an increase in host species diversity reduces disease levels, due to a lower realized host density. Finally, if pathogen species abundance distributions in this community have a long tail of rare pathogen species, our sample size would need to be much larger in order to observe many of these pathogen species on multiple individual plants in the community and thus document their more generalized host specificity patterns (Rottstock et al., 2014).

Exotic plants in our study did not show lower diversity or infection levels and instead seem susceptible to a broad range of pathogens. This finding differs from other studies showing that plants escape foliar pathogens in their naturalized range (Mitchell & Power, 2003) or that introduced plants experience reduced attack by fungal and viral pathogens, compared to native plants (Agrawal et al., 2005). However, these studies focused mainly on obligate, biotrophic fungi, which display a high host specificity and are hence rather unlikely to be co‐introduced with their host (Parker & Gilbert, 2004). In contrast, the pathogen community infecting exotic hosts in our study were mostly generalist necrotrophic and hemibiotrophic species that are globally distributed and are found in a broad range of ecosystems (see also Parker & Gilbert, 2007). We find that pathogens infecting exotic hosts had broader host ranges than those infecting native hosts, in accordance with our expectation (see also Blaisdell & Roy, 2014).

The lack of differences between native and exotic host species in our study is a robust result, as we have good statistical power (see Table 2), and several exotic and native plants were found to have high diversity and infection of pathogens (e.g., exotic *Melilotus albus* had the highest level of pathogen severity of all sampled plant species). This indicates one or more of the following mechanisms: (a) Hosts are introduced with their pathogens to new continents, (b) enemy release is transitory, and natural enemies accumulate with time since introduction (Brändle, Kühn, Klotz, Belle, & Brandl, 2008; Mordecai, 2011), and/or (c) that native pathogens effectively infect exotic plants (Elton, 1958). Our study was not designed to distinguish between these mechanisms but, as many of the pathogens in our system have a global distribution (see Table 4), our results support the first and/or the third option. We also found one case of a native pathogen, only reported from North America, infecting an exotic species (*Phyllachora lespedezae* on *Kummerowia stipulacea*). An increasing time since first introduction (ca. 300 years in the case of *Melilotus albus*), as well as multiple introduction events (Dutech, Fabreguettes, Capdevielle, & Robin, 2010), might additionally contribute to a high pathogen load. We note that few of our exotic plant species are considered invasive (only *Lespedeza cuneata* and *Melilotus albus*, according to the Missouri Department of Conservation, https://nature.mdc.mo.gov/status/invasive), and it is possible that communities with more invasive exotic plants might show more support for the enemy release hypothesis (Mitchell & Power, 2003). Finally, anthropogenic disturbance might override the importance of enemy release (Colautti et al., 2004; Elton, 1958; Hierro, Villarreal, Eren, Graham, & Callaway, 2006; Lozon & MacIsaac, 1997; St. Clair,  O’Connor, Gill & McMillan,^2016^) for exotic plants in our system. Grazing by deer (*Odocoileus virginianus*) and management using mowing and prescribed fires are common at our study sites and may create niche opportunities for exotic plants.

Our statistical analysis approach tested whether abundance and origin influenced pathogen infection using data pooled into broad groups (i.e., our pathogen groups). This approach gives us more power to test for the relationship than if we had used species‐level identifications of pathogens, which we mostly have for all pathogen groups except viruses and bacteria. For our test of host abundance, specialization is the precondition that by which there is a relationship between host abundance and infection (Mordecai, 2011; Spear & Mordecai, 2018). If host abundance plays a role in infection for many specialized pathogens, then there would be greater ability to detect this in an analysis that combines many specialized pathogen species together in a single analysis. Likewise, if native species are infected by more specialized pathogens than exotics species, we expect to be better able to detect a significant effect of origin on pathogen infection in an analysis that considers pathogen groups.

We found no phylogenetic signal across plants in the diversity and infection of pathogens. This is surprising, given the role of phylogenetic signal found in other community‐level studies (e.g., Parker et al., 2015). However, there is evidence in other systems that both pathogen virulence and susceptibility to pathogens and herbivores are more dependent on local selective conditions than evolutionary history (Agrawal & Fishbein, 2006; Haak, Ballenger, & Moyle, 2014; Rudgers, Strauss, & Wendel, 2004; Thrall, Burdon, & Bever, 2002). For example, heterogeneous landscapes (such as the matrix of forest and grassland habitats in our system) can favor rapid local adaption of both host and pathogen populations (Forester, Jones, Joost, Landguth, & Lasky, 2016), creating unique patterns of host susceptibility and pathogen virulence (Thrall et al., 2002) that weaken phylogenetic signals across hosts in pathogen infection levels (Haak et al., 2014; Strauss, Rudgers, Lau, & Irwin, 2002).

In conclusion, our study suggests that neither host abundance nor origin or evolutionary relationship play an important role in explaining general patterns of pathogen infection in our studied grassland communities. Our broad methodology provides a guide that could be applied to other ecosystems, so that the generality of this result and/or its context dependence (e.g., we consider relatively disturbed temperate grasslands) could be synthesized in the future. Our study could be expanded by considering experiments that manipulate host abundance, by replicating observations across years to examine temporal variability of results (see Rottstock et al., 2014), and by designing observational and experimental studies to test among underlying mechanisms (Hector et al., 2007). Studies such as this one, which consider multiple factors and multiple pathogen groups, are critical to understanding the role of pathogens in mediating community dynamics.

## CONFLICT OF INTEREST

The authors declare that they have no conflict of interest.

## AUTHOR CONTRIBUTION

**Robin Schmidt:** Conceptualization (equal); Data curation (lead); Formal analysis (equal); Investigation (lead); Methodology (equal); Visualization (lead); Writing‐original draft (lead); Writing‐review & editing (equal). **Harald Auge:** Conceptualization (equal); Formal analysis (equal); Funding acquisition (lead); Methodology (equal); Supervision (supporting); Writing‐original draft (equal); Writing‐review & editing (supporting). **Holger B. Deising:** Funding acquisition (supporting); Methodology (supporting); Supervision (supporting); Writing‐original draft (supporting); Writing‐review & editing (supporting). **Isabell Hensen:** Funding acquisition (equal); Supervision (supporting); Writing‐review & editing (supporting). **Scott A. Mangan:** Resources (equal); Supervision (supporting); Writing‐review & editing (supporting). **Martin Schädler:** Conceptualization (supporting); Formal analysis (supporting); Funding acquisition (supporting); Supervision (supporting); Writing‐review & editing (supporting). **Claudia Stein:** Resources (equal); Supervision (supporting); Writing‐review & editing (supporting). **Tiffany M. Knight:** Conceptualization (lead); Data curation (supporting); Formal analysis (equal); Investigation (supporting); Methodology (equal); Resources (equal); Supervision (lead); Visualization (supporting); Writing‐original draft (equal); Writing‐review & editing (equal).

## Supporting information

Supplementary Material

Supplementary Material

## Data Availability

Data associated with this article are available in the Dryad Digital Repository: https://doi:10.5061/dryad.zkh189372
