# Supplementary material for: Abundance, origin, and phylogeny of plants do not predict community‐level patterns of pathogen diversity and infection
Source: Ecol Evol. 2020 May 18;10(12):5506–16. doi: 10.1002/ece3.6292 (PMC7319236; doi:10.1002/ece3.6292)
Supplement: Supplementary file 1 — Supplementary Material [file ECE3-10-5506-s001.docx]

**Appendices**

Appendix A1. Plant species at the ‘Perilla site’, along with their origin status (N=native, E=exotic), abundance (average percent cover) and presence or absence of infection by different pathogen groups (X indicates presence). B/V = Bacterial and viral diseases.

|  |  |  |  | **Infection by** | | | | |
| --- | --- | --- | --- | --- | --- | --- | --- | --- |
| **Plant species** | **Family** | **Origin** | **Abundance [%]** | **Downy mildews** | **Fungal leaf spot diseases** | **Powdery mildews** | **Rusts** | **B/V diseases** |
| *Ageratina altissima* | Asteraceae | N | 2.4 |  | X |  |  | X |
| *Carex muhlenbergii* | Cyperaceae | N | 11.4 |  |  |  | X | X |
| *Croton monanthogynus* | Euphorbiaceae | N | 0.1 |  |  |  | X |  |
| *Daucus carota* | Apiaceae | E | 0.1 |  | X |  |  | X |
| *Desmodium paniculatum* | Fabaceae | N | 0.1 |  |  |  |  | X |
| *Festuca subverticillata* | Poaceae | N | 3.7 |  | X | X |  | X |
| *Galium tinctorium* | Rubiaceae | N | 0.3 |  | X |  |  | X |
| *Geum canadense* | Rosaceae | N | 3.5 |  | X |  |  | X |
| *Muhlenbergia sobolifera* | Poaceae | N | 0.8 |  | X |  |  | X |
| *Oxalis stricta* | Oxalidaceae | N | 0.1 |  | X |  |  |  |
| *Parietaria pennsylvanica* | Urticaceae | N | 11.5 |  |  |  |  | X |
| *Perilla frutescens* | Lamiaceae | E | 9.8 |  |  |  |  | X |
| *Pilea pumila* | Urticaceae | N | 0.1 |  |  |  |  |  |
| *Plantago lanceolata* | Plantaginaceae | E | 0.1 |  |  |  |  | X |
| *Plantago rugelli* | Plantaginaceae | N | 0.2 |  |  |  |  | X |
| *Polygonum pennsylvanicum* | Polygonaceae | N | 0.9 |  |  |  |  |  |
| *Torilis arvensis* | Apiaceae | E | 0.1 |  | X |  |  | X |
| *Viola sororia* | Violaceae | N | 0.6 |  |  |  |  | X |

Appendix A2. Plant species at the ‘Carduus site’, along with their origin status (N=native, E=exotic), abundance (average percent cover) and presence or absence of infection by different pathogen groups (X indicates presence). B/V = Bacterial and viral diseases.

|  |  |  |  | **Infection by** | | | | |
| --- | --- | --- | --- | --- | --- | --- | --- | --- |
| **Plant species** | **Family** | **Origin** | **Abundance [%]** | **Downy mildews** | **Fungal leaf spot diseases** | **Powdery mildews** | **Rusts** | **Bacterial & viral diseases** |
| *Calystegia sepium* | Convolvulaceae | N | 1.6 |  |  |  | X | X |
| *Cirsium discolor* | Asteraceae | N | 0.2 |  | X |  |  |  |
| *Conyza canadensis* | Asteraceae | N | 6.1 |  |  |  |  | X |
| *Croton monanthogynus* | Euphorbiaceae | N | 3.3 |  |  |  | X | X |
| *Daucus carota* | Apiaceae | E | 1.1 |  |  | X |  | X |
| *Desmodium canadense* | Fabaceae | N | 0.6 |  |  |  |  | X |
| *Elephantopus caroliniana* | Asteraceae | N | 2.3 |  | X |  |  |  |
| *Eupatorium altissimum* | Asteraceae | N | 0.2 |  | X |  |  |  |
| *Eupatorium serotinum* | Asteraceae | N | 0.1 |  |  |  |  |  |
| *Geum canadense* | Rosaceae | N | 0.1 |  |  |  |  |  |
| *Helianthus pauciflorus* | Asteraceae | N | 1.6 |  |  |  |  | X |
| *Lespedeza cuneata* | Fabaceae | E | 7.0 |  |  |  |  | X |
| *Monarda fistulosa* | Lamiaceae | N | 0.2 |  | X |  |  |  |
| *Oxalis stricta* | Oxalidaceae | N | 0.8 |  |  |  |  | X |
| *Phyla lanceolata* | Verbenaceae | N | 1.6 |  | X |  |  | X |
| *Plantago rugelli* | Plantaginaceae | N | 0.1 |  | X |  |  | X |
| *Polygonum aviculare* | Polygonaceae | E | 0.3 |  |  |  |  |  |
| *Solanum carolinense* | Solanaceae | N | 7.7 |  | X |  |  | X |
| *Taraxacum officinale* | Asteraceae | E | 2.4 |  |  |  |  |  |
| *Teucrium canadense* | Lamiaceae | N | 2.0 |  | X |  |  |  |
| *Verbena urticifolia* | Verbenaceae | N | 0.3 |  | X |  |  | X |
| *Verbesina alternifolia* | Asteraceae | N | 4.0 |  | X |  |  | X |
| *Vernonia baldwinii* | Asteraceae | N | 0.5 |  |  |  | X | X |

Appendix A3. Plant species at the ‘Potentilla site’, along with their origin status (N=native, E=exotic), abundance (average percent cover) and presence or absence of infection by different pathogen groups (X indicates presence). B/V = Bacterial and viral diseases.

|  |  |  |  | **Infection by** | | | | |
| --- | --- | --- | --- | --- | --- | --- | --- | --- |
| **Plant species** | **Family** | **Origin** | **Abundance [%]** | **Downy mildews** | **Fungal leaf spot diseases** | **Powdery mildews** | **Rusts** | **B/V diseases** |
| *Acalypha virginica* | Euphorbiaceae | N | 0.2 |  | X |  |  |  |
| *Ambrosia artemisiifolia* | Asteraceae | N | 0.3 |  |  |  |  | X |
| *Coreopsis lanceolata* | Asteraceae | N | 0.1 |  |  |  |  | X |
| *Croton monanthogynus* | Euphorbiaceae | N | 4.1 |  |  |  | X |  |
| *Erigeron annuus* | Asteraceae | N | 6.6 |  | X |  | X | X |
| *Eupatorium altissimum* | Asteraceae | N | 0.1 |  | X |  |  |  |
| *Euphorbia dentata* | Euphorbiaceae | N | 0.5 |  |  |  |  | X |
| *Festuca subverticillata* | Poaceae | N | 11.4 | X |  |  |  | X |
| *Kummerowia stipulacea* | Fabaceae | E | 0.5 |  | X |  |  | X |
| *Lespedeza cuneata* | Fabaceae | E | 0.4 |  |  |  |  | X |
| *Medicago lupulina* | Fabaceae | E | 2.3 |  | X | X |  | X |
| *Melilotus albus* | Fabaceae | E | 0.3 |  | X |  | X | X |
| *Potentilla recta* | Rosaceae | E | 6.6 |  | X |  |  |  |
| *Ratibida pinnata* | Asteraceae | N | 0.3 |  |  |  |  | X |
| *Ruellia humilis* | Acanthaceae | N | 4.6 |  |  |  | X |  |
| *Senna marilandica* | Fabaceae | N | 0.2 |  | X |  |  |  |
| *Stenaria nigricans* | Rubiaceae | N | 0.9 |  | X |  |  |  |
| *Symphyotrichum oblongifolium* | Asteraceae | N | 1.0 |  |  |  |  | X |
| *Teucrium canadense* | Lamiaceae | N | 2.2 |  |  |  |  | X |
| *Trichostema brachiata* | Lamiaceae | N | 0.1 |  | X |  |  |  |

Appendix B. Geographic distribution, global host ranges and infected (bold) and known, but not infected host species for each pathogen species at the three study sites. Asterisks (*****) indicate pathogen-host combinations not yet reported for the US (according to the **USDA Fungus-Host distribution database). PM = Powdery mildews. NA = North America, EU = Europe, AS = Asia, SA = South America, AF = Africa, AU = Australia and Oceania.**

|  | **Pathogen**  (Global host range in  number of genera and families) | **Geographic distribution** | **Host species per site**  Perilla site Carduus site Potentilla site | | |
| --- | --- | --- | --- | --- | --- |
| Fungal leaf spot diseases | *Ascochyta caulicola* (5; 1) | NA, EU, AS |  |  | ***M***. ***albus*** |
|  | *Boeremia exigua* (275; 83) | cosmopolitan | *P*. *lanceolata* | ***S***. ***carolinense***,  ***T***. ***canadense***, *D. carota*,  *G*. *canadense*, *M. fistulosa*, *P. rugelli*, *T. officinale* |  |
|  | *Cercospora apii* (241; 79) | cosmopolitan | ***T***. ***arvensis****, *D*. *carota*,  *P*. *lanceolata* | *D*. *carota* | *P*. *recta* |
|  | *Cercospora elephantopi* (2; 1) | NA, SA, AF |  | ***E***. ***carolinianus*** |  |
|  | *Cercospora galii* (1; 1) | NA, EU, AS | ***G***. ***tinctorium*** |  |  |
|  | *Cercospora houstoniae* (2; 1) | NA |  |  | ***S***. ***nigricans*** |
|  | *Cercospora isanthi* (1; 1) | NA |  |  | ***T***. ***brachiata*** |
|  | *Cercospora medicaginis* (6; 3) | cosmopolitan |  |  | ***M***. ***lupulina***, *M*. *albus* |
|  | *Colletotrichum dematium* (206; 76) | cosmopolitan | ***O***. ***stricta****,  *P*. *pennsylvanicum* | *P*. *aviculare* |  |
|  | *Leptosphaeria muehlenbergiae* (1; 1) | NA | ***M***. ***sobolifera*** |  |  |
|  | *Phyllachora lespedezae* (4; 1) | NA |  |  | ***K***. ***stipulacea*** |
|  | *Septoria* cf*. mississippiensis* (1; 1) | NA | ***M***. ***sobolifera**** |  |  |
|  | *Septoria erigerontis* (3; 1) | NA, EU, AS, AU, SA |  | *C*. *canadensis* | ***E***. ***annuus*** |
|  | *Septoria gei* (1; 1) | NA, EU, AS | ***G***. ***canadense*** |  |  |
|  | *Septoria verbenae* (1; 1) | NA, SA, EU, AU |  | ***V***. ***urticifolia*** |  |
| PM | *Erysiphe heraclei* (91; 7) | cosmopolitan | ***D***. ***carota***,  *T*. *arvensis* |  |  |
|  | *Erysiphe pisi* (293; 75) | cosmopolitan |  | *P*. *aviculare* | ***M***. ***lupulina***, *L*. *cuneata*,  *K*. *stipulacea*, *M*. *albus* |
| Rusts | *Coleosporium vernoniae* (6; 2) | NA, SA, AS |  | ***V***. ***baldwinii***, *E*. *carolinianus* |  |
|  | *Phakopsora crotonis* (3; 1) | NA, SA | ***C***. ***monanthogynus*** | ***C***. ***monanthogynus*** | ***C***. ***monanthogynus*** |
|  | *Puccinia convolvuli* (6; 1) | cosmopolitan |  | ***C***. ***sepium*** |  |
|  | *Puccinia dioicae* (40; 9) | NA, EU, AS, SA | ***C***. ***muehlenbergii*** |  | *E*. *annuus* |
|  | *Puccinia extensicola* (43; 8) | NA, EU, AS, SA | *C*. *muehlenbergii* |  | ***E***. ***annuus*** |
|  | *Puccinia lateripes* (9; 2) | NA, SA |  |  | ***R. humilis*** |
|  | *Uromyces striatus* (17; 2) | cosmopolitan |  |  | ***M. albus***, *M*. *lupulina* |
