# Supplementary figures and images for: Abundance, origin, and phylogeny of plants do not predict community‐level patterns of pathogen diversity and infection
Source: Ecol Evol. 2020 May 18;10(12):5506–16. doi: 10.1002/ece3.6292 (PMC7319236; doi:10.1002/ece3.6292)

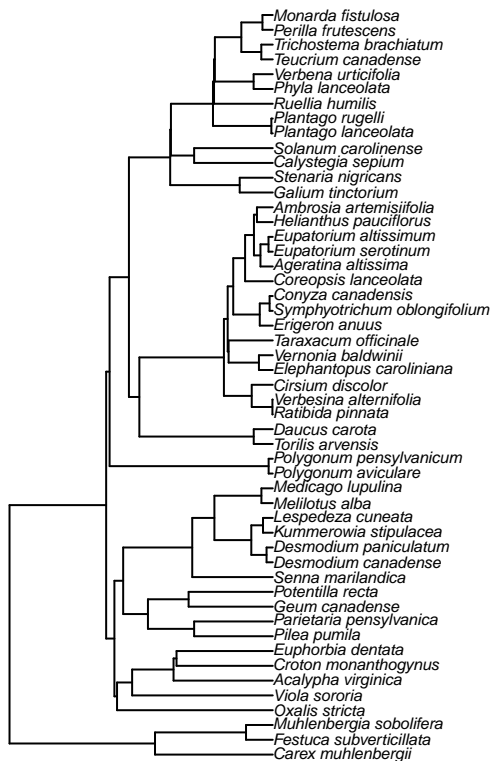

Supplement: Supplementary file 2 — Supplementary Material [file ECE3-10-5506-s002.pdf]
